# Crosslinked Carbon Nanotubes/Polyaniline Composites as a Pseudocapacitive Material with High Cycling Stability

**DOI:** 10.3390/nano5021034

**Published:** 2015-06-11

**Authors:** Dong Liu, Xue Wang, Jinxing Deng, Chenglong Zhou, Jinshan Guo, Peng Liu

**Affiliations:** State Key Laboratory of Applied Organic Chemistry and Key Laboratory of Nonferrous Metal Chemistry and Resources Utilization of Gansu Province, College of Chemistry and Chemical Engineering, Lanzhou University, Lanzhou 730000, China; E-Mails: liud13@lzu.edu.cn (D.L.); wangx2012@lzu.edu.cn (X.W.); dengjx12@lzu.edu.cn (J.D.); zhoucl11@lzu.edu.cn (C.Z.); gjs@lzu.edu.cn (J.G.)

**Keywords:** electrochemical energy storage, cycling stability, nanocomposite, crosslinked carbon nanotubes (C-CNTs), polyaniline (PANI)

## Abstract

The poor cycling stability of polyaniline (PANI) limits its practical application as a pseudocapacitive material due to the volume change during the charge-discharge procedure. Herein, crosslinked carbon nanotubes/polyaniline (C-CNTs/PANI) composites had been designed by the *in situ* chemical oxidative polymerization of aniline in the presence of crosslinked carbon nanotubes (C-CNTs), which were obtained by coupling of the functionalized carbon nanotubes with 1,4-benzoquinone. The composite showed a specific capacitance of 294 F/g at the scan rate of 10 mV/s, and could retain 95% of its initial specific capacitance after 1000 CV cycles. Such high electrochemical cycling stability resulting from the crosslinked skeleton of the C-CNTs makes them potential electrode materials for a supercapacitor.

## 1. Introduction

With the rise in environmental pollution and the rapid growth of portable electronics, high-efficiency energy storage devices such as supercapacitors have become the most promising vehicles in the last decades. Supercapacitors, also called electrochemical capacitors, are a kind of high-performance energy storage devices possessing a long life cycle and high power density [1]. They can be broadly classified into two categories, electrochemical double layer capacitors (EDLCs) and pseudocapacitors [2]. For the EDLCS, the energy storage depends on the separation of charges at the electrode–electrolyte interface [3]; on the other hand, the pseudocapacitors are based on the Faradaic reaction, which is essentially a redox reaction occurring between the surface of electrode material and electrolyte [4]. Notably, the pseudocapacitors open an opportunity to increase the energy density [3].

In the past decades, transition metal oxides [5] and electrically conducting polymers [6] have been extensively applied for supercapacitors as pseudocapacitive materials. Although these pseudocapacitive materials suffer from low electrical conductivity and poor stability, the electrically conducting polymers offer both good electric conductivity and excellent pseudocapacitive behaviors compared to the transition metal oxides [7]. Among the electrically conducting polymers, polyaniline (PANI) has attracted more interest due to its advantages over the others, such as easy synthesis, low cost, good processability, high environment stability, and reversible control of electrical properties by both charge-transfer doping and protonation [8]. However, as a potential pseudocapacitive material, it still suffers from limited cycling stability, high self-discharge rate, and low attainable doping degree as well as mass transport limitation within thick polymer layers [9]. To resolve these issues, PANI has been crosslinked [10], nano-structured [11], or supported on various inorganic nanomaterials as composites [12].

Carbon nanotubes (CNTs) are one of the most used supporting materials for PANI, created by coating PANI on its surface as core/shell nanocomposites in order to improve the cycling stability of PANI. With the pristine CNTs as support, the cycling stability of PANI is far from desirable [13,14], although it was higher than the pure PANI materials. However, the specific capacitance retention could achieve about 90% when the functionalized CNTs were used [15,16], mainly due to the interaction between PANI and the functionalized CNTs. Hyder and co-workers synthesized the polyaniline nanofiber/MCNT film via layer-by-layer assembly technique, which could retain 96% of its initial specific capacitance after 1000 cycles [17], but the process is much more complicated than the facile *in* *situ* polymerization technique.

It has been reported that enhanced electrical conductivity and functionalities could be achieved for the pseudocapacitive materials by depositing them on a highly conductive network (such as a carbon-based material) [18]. Furthermore, the charge diffusion length is shortened, and therefore can improve the power density of the device [7]. A three-dimensional crosslinked carbon network (3D-CCN), created by carbonizing a commercial macroporous melamine sponge, was used as a contact-resistance-free substrate for PANI-based pseudo-supercapacitor [19]. The electrodes showed a small decrease and retained up to 83.2% of their original specific capacitance value after 1000 cycles. In the system, the highly conductive 3-D network was advantageous for supporting facile charge transfer and rapid charge gain/loss in the active materials even at a high current density. In addition, the thin coating of PANI and the spaces between the nanowires ensured full contact between the electrolyte and PANI nanowires, thus resulting in a high utilization rate for PANI. Therefore, the rate performance and capacitance were considerably improved, compared to those of many other PANI/carbon composite electrodes.

Liu *et al.* [20] designed a 3-D graphene/PANI composite for a supercapacitor via the *in* *situ* polymerization of aniline in the presence of 3-D graphene suspension, in which the 3-D graphene was obtained by heating the graphene oxide under vacuum. After 500 cycles, the capacity decay was only 9.4% of its initial discharge capacity. This indicated that the 3-D nanoporous structure may prevent polymer swelling and shrinking more effectively, thus resulting in better cyclic life. A 3-D graphene framework, prepared via chemical vapor deposition (CVD), has also been used for the chemically grown PANI nanofibers. Its capacity retention was found to be ~86.5% after 5000 continuous charge-discharge cycles [21]. Zhong *et al.* [7] prepared 3-D highly porous CNT-based sponges via thermal CVD as a conductive substrate for the deposition of PANI. Their capacitance showed less than 2% decay after 3000 cycles.

**Scheme 1:**
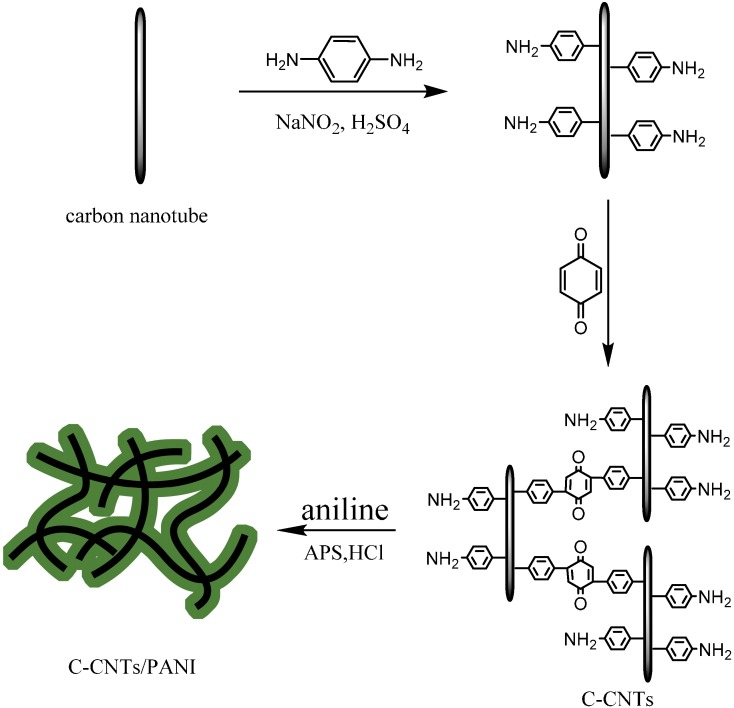
Schematic illustration of the fabrication of the crosslinked carbon nanotubes (C-CNTs) and C-CNTs/polyaniline (PANI) composites.

In the present work, we developed a simple way to synthesize 3-D crosslinked carbon nanotubes (C-CNTs)/PANI pseudocapacitive materials (Scheme 1). Firstly, pristine commercial carbon nanotubes were covalently functionalized via a diazonium reaction and then the diazonium salt of the CNTs was crosslinked via the coupling of 1,4-benzoquinone [22]. Subsequently, PANI was coated onto the 3-D framework of the C-CNTs via the facile *in situ* chemical oxidative polymerization of aniline. The effect of the feeding ratios of the C-CNTs to aniline on the morphology, thermal stability, and electrochemical properties of the 3-D C-CNTs/PANI pseudocapacitive materials was investigated.

## 2. Results and Discussion

### 2.1. C-CNTs

**Figure 1:**
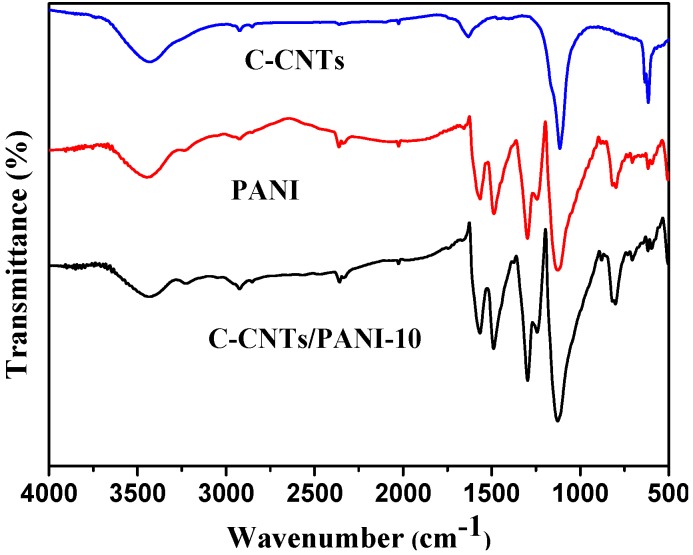
Fourier transform infrared (FT-IR) spectra of the C-CNTs, pure PANI, and the C-CNTs/PANI-10 composite.

The C-CNTs were synthesized from commercial multi-wall carbon nanotubes via coupling their diazonium salts with 1,4-benzoquinone, as shown in Scheme 1. In the Fourier transform infrared (FT-IR) spectrum of the product (Figure 1), the bands at approximate 1297 cm^−1^ and 1243 cm^−1^ demonstrate the presence of C–N stretching mode of the aromatic amine [10]. The peak at 1630 cm^−1^ is attributed to the C=O groups in benzoquinone, indicating the successful crosslinking of the pristine CNTs via the diazotization-coupling reaction with 1,4-benzoquinone [22].

**Figure 2:**
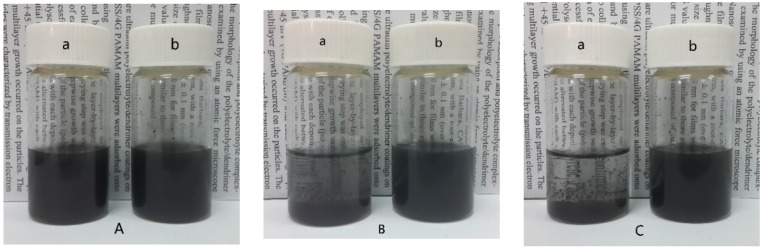
Digital photos of the ethanol deposition of the C-CNTs (a) and pristine CNTs (b) after standing for different times: (**A**) 0 h; (**B**) 6 h; and (**C**) 12 h.

To confirm the crosslinking of the carbon nanotubes, the product or the pristine multi-wall carbon nanotubes were dispersed into ethanol with a concentration of 2.0 mg/mL by ultrasonication for 5 min. No obvious sediment could be seen from the dispersion of the pristine CNTs after standing for 12 h, while most of the C-CNTs subsided as naked-eye visible matter from the dispersion even after 6 h (Figure 2). This demonstrated that the C-CNTs showed poorer dispersion stability than the pristine CNTs, although there were polar organic groups on their surfaces. The results revealed that the carbon nanotubes had been crosslinked with each other to successfully form the micrometer-scaled 3-D framework.

**Figure 3:**
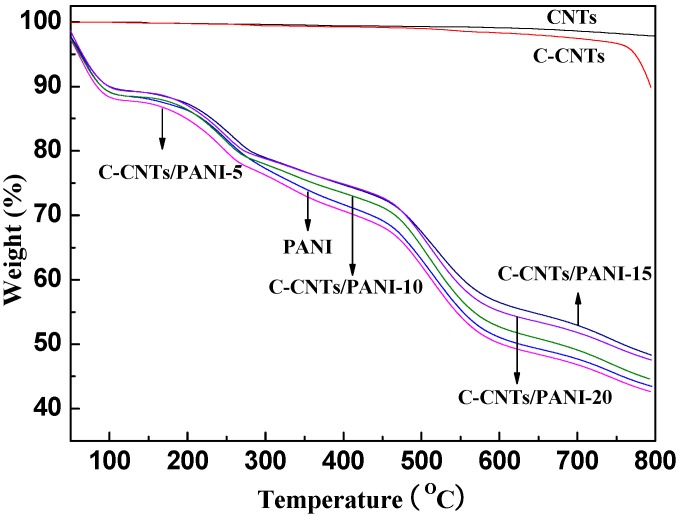
TGA curves of the pristine CNTs, C-CNTs, pure PANI, and C-CNTs/PANI composites.

Compared with the high thermal stability of the pristine CNTs (Figure 3), the weight loss of the C-CNTs at the temperature range of 500–750 °C is due to the decomposition of the organic crosslinking groups [22,23]. Furthermore, the obvious weight loss starting from 750 °C is caused by the thermal decomposition of the defects (C–C bond) of the C-CNTs caused by the diazonium reaction [24].

### 2.2. C-CNTs/PANI Composites

**Figure 4:**
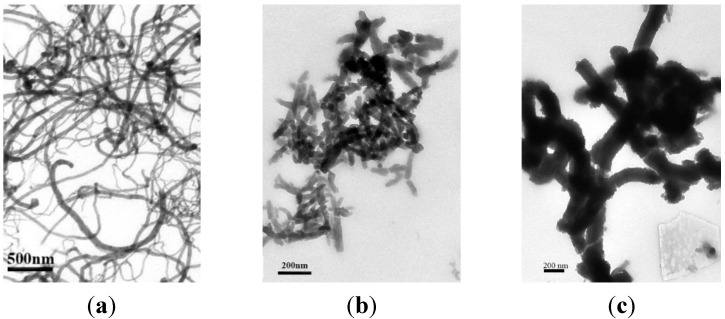

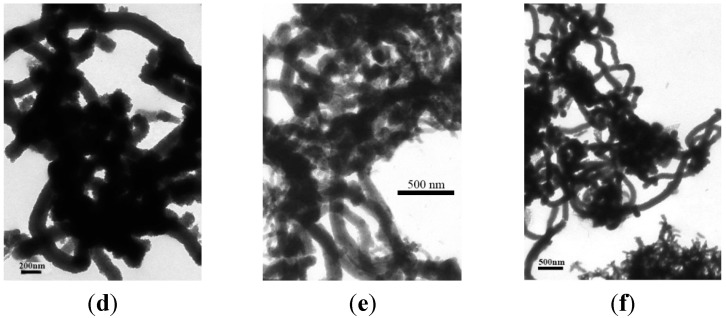
Transmission electron microscopy (TEM) images of (**a**) C-CNTs; (**b**) Pure PANI; (**c**) C-CNTs/PANI-5; (**d**) C-CNTs/PANI-10; (**e**) C-CNTs/PANI-15; and (**f**) C-CNTs/PANI-20.

Next PANI was coated onto the 3-D framework of the crosslinked carbon nanotubes (C-CNTs) via *in situ* chemical oxidative polymerization of aniline. The morphology of the C-CNTs, pure PANI, and C-CNTs/PANI composites prepared with the different C-CNTs/aniline feeding ratios were compared with the TEM technique (Figure 4). Without C-CNTs, short PANI nanorods were produced. In the presence of the C-CNTs, aniline was adsorbed onto the C-CNTs via π-π * electron interaction and hydrogen bond [25], and polymerized onto the C-CNTs to form a core/shell structure, in which some PANI chains might be grafted onto the C-CNTs via the copolymerization of an aniline monomer with the aminophenyl groups on the C-CNTs [26]. With an increase in the feeding ratio of C-CNTs to aniline from 0.05:1 to 0.2:1, the diameter of the core/shell PANI-coated CNTs decreased from 150 nm to 100 nm.

In the FT-IR spectrum of the products (Figure 1), the C–N stretching vibrations in the polaronic structure located at 1240 cm^−1^ position [27], and the peaks at 1565 and 1487 cm^−1^ confirm the existence of the C=C stretching of quinoid and benzenoid ring vibrations, respectively [28], revealing the successful coating of PANI. The absorbance at 1140 cm^−1^ corresponds to the aromatic C–H structure in both C-CNTs and PANI [29]. The stronger absorbance at 1140 cm^−1^ of the C-CNTs/PANI nanocomposite than the pure PANI also reveal the successful preparation of the nanocomposite. The absorbance peak at 809 cm^−1^ and the smaller peak at 869 cm^−1^ correspond to the out-of-plane C-H motion [30]. Figure 3 also shows the TGA curves of the C-CNTs/PANI composites prepared with the different feeding ratio of C-CNTs to aniline—a typical three-step weight loss for PANI. The small fraction of weight loss below 100 °C arises mainly from the expulsion of moisture or ethanol and the free acid trapped in the PANI. The second step for weight loss at 150–250 °C is due to the loss of acid dopant. Then a very significant weight loss occurs at about 450 °C due to thermal decomposition of the polymer chain [31].

**Figure 5:**
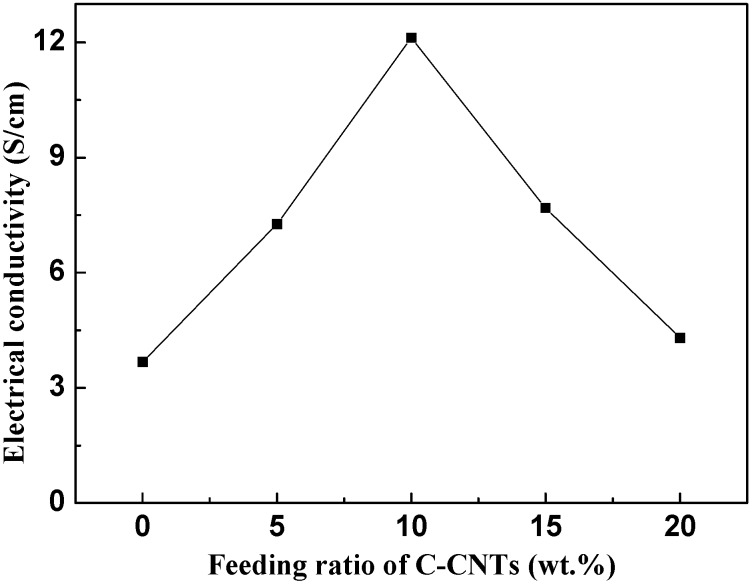
Electrical conductivity of the pure PANI and C-CNTs/PANI composites.

Figure 5 shows the electrical conductivity of the C-CNTs/PANI composites with different feeding ratios of C-CNTs, measured with the standard four probes method at room temperature. The electrical conductivity has a significant influence on the C-rate performance, not only decreasing the impedance of the electrode, but also helping the electron transfer inside the material [32]. It is noteworthy that all the electrical conductivity values of the C-CNTs/PANI composites were higher than for pure PANI. When the feeding ratio of the C-CNTs was lower than 10 wt%, the conductivity of the composites gradually increased, reaching the highest electrical conductivity of 12.12 S/cm for the C-CNTs/PANI-10 composite, because the electrons could transport through the overlapped PANI-PANI contact between the C-CNT/PANI bundles [30]. However, when the feeding ratio of the C-CNTs increased further, the conductivity of the composites showed a downward trend; this was mainly due to the diameter of PANI-coated C-CNTs decreasing (Figure 3c–f) and the contact areas between C-CNT/PANI bundles decreasing. The relatively high electrical conductivity of the C-CNTs/PANI composites in the present work might be mainly due to the fact that the C-CNTs may serve as a “conducting bridge” between the PANI-conducting domains, thus increasing the effective path [27].

### 2.3. Electrochemical Performances

Due to their highly conductive 3-D network, the electrochemical performance of the CNTs/PANI composites were characterized by galvanostatic charge/discharge (GCD), cyclic voltammetry (CV), and cycling stability measurements.

**Figure 6:**
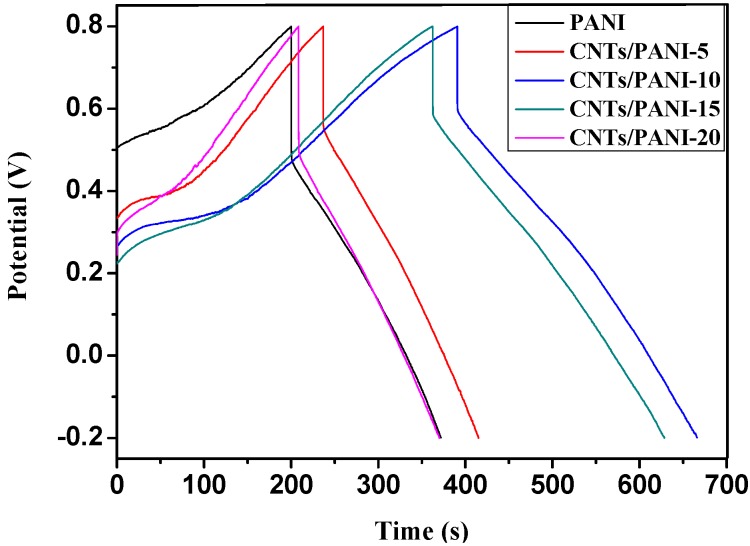
Galvanostatic charge/discharge (GCD) curves of the C-CNTs/PANI composite electrodes at a current density of 1 A/g in 1.0 M H_2_SO_4_ solution.

The GCD curves of the C-CNTs/PANI composite electrodes were compared with the pure PANI electrode in order to further investigate their pseudocapacitive behavior, at a current density of 1A/g in the potential range from −0.2 to 0.8 V in 1.0 mol/L H_2_SO_4_ aqueous electrolytes (Figure 6). The GCD curves were not straight, indicating the occurrence of a Faradaic reaction among the electrode materials [33]. It was observed that the specific capacitance of the samples increased from 171.7 F/g of the PANI electrode to the maximum specific capacitance of 275.4 F/g of the C-CNTs/PANI-10 composite electrode with the C-CNTs feeding ratio of 10 wt.%, and then decreased to 161.5 F/g of the C-CNTs/PANI-20 composite electrode with the C-CNTs feeding ratio of 20 wt.%, a similar trend to the electrical conductivity.

**Figure 7:**
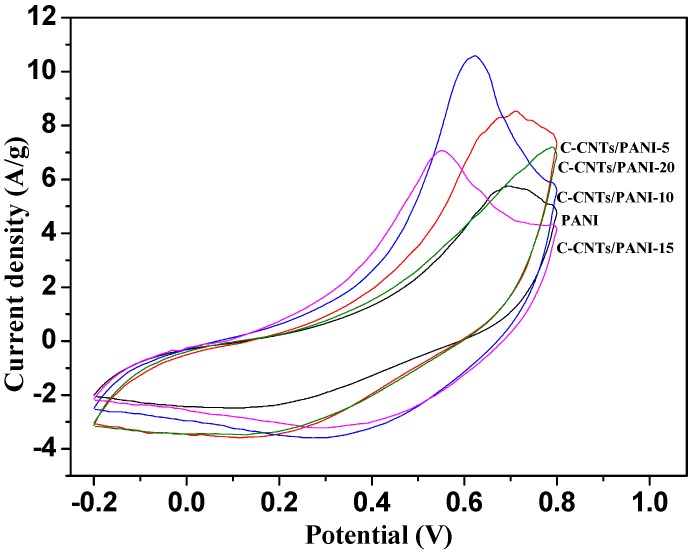
Cyclic voltammetry (CV) curves of the C-CNTs/PANI composites electrodes at scan rates of 10 mV/s.

Figure 7 presents the CV behavior of the C-CNTs/PANI composite electrodes prepared with different feeding ratios of C-CNTs to PANI at a scan rate of 10 mV/s in 1.0 mol/L aqueous H_2_SO_4_ solution. The redox peaks could be easily seen in the CV curves, demonstrating the pseudocapacitance behavior of the composites. Here, an accurate specific capacitance (*C_m_*) could be obtained from the surrounding areas of CV curves. With the increase of the feeding ratio of the C-CNTs, the *C_m_* values were 283.2 F/g, 298.4 F/g, 251.2 F/g, and 237.5 F/g for the C-CNTs/PANI-5, C-CNTs/PANI-10, C-CNTs/PANI-15, and C-CNTs/PANI-20 composite electrodes, respectively. All the *C_m_* values were higher than that of the pure PANI of 187.8 F/g. The results indicated that the 3-D C-CNTs framework was favorable to the electrochemical energy storage of PANI.

**Figure 8:**
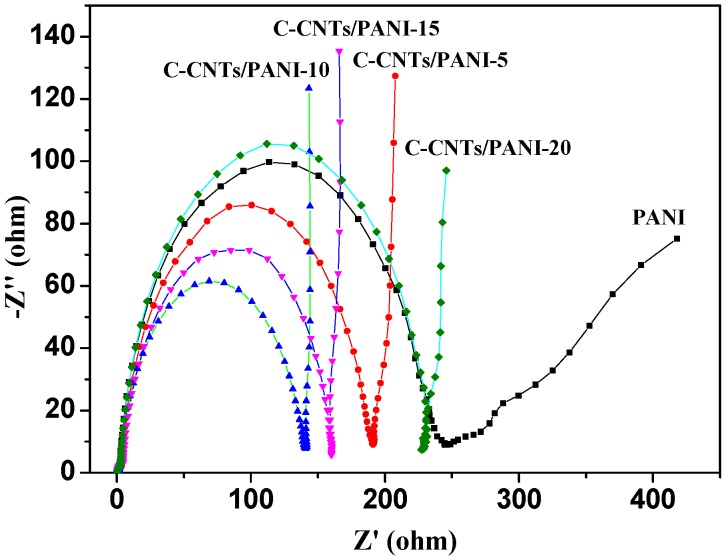
Nyquist plots of the C-CNTs/PANI composite electrodes.

The electrochemical impedance spectrum (EIS) of the C-CNTs/PANI composite electrodes were carried out at an open circuit potential of 0.4 V over a frequency range of 10^−2^–10^5^ Hz, in order to investigate the mass and charge transfer behavior at electrode interface. It can be observed from Figure 8 that all the Nyquist plots included two distinctive parts, which were straight lines in the low frequency region and semicircular arcs in the high frequency region [34]. The C-CNTs/PANI composite electrodes showed approximately vertical lines in the low frequency region, indicating lower ion diffusion resistance and better capacitive behaviors than the PANI electrode which showed a straight line with slope of 45. This was mainly due to the fact that the more vertical the curve is, the more closely the supercapacitor behaves as an ideal capacitor [35]. The semicircular arcs in high frequency regions indicated charge transfer resistance (*R_ct_*) caused by the electrochemical reactions at the contact interface between the electrode and the electrolyte solution [36,37]. The *R_ct_* value of the PANI was measured to be 245.7 Ω. The decreased *R_ct_* values for the C-CNTs/PANI composite electrodes with the same changing trend as their electrical conductivity demonstrated the high charge transfer rate. It could also be confirmed by observing the negligible voltage drop at the beginning of discharge curves in Figure 6. However, their *R_ct_* values were higher than those free-standing electrode materials [7,36], maybe due to their micro-scaled size.

**Figure 9:**
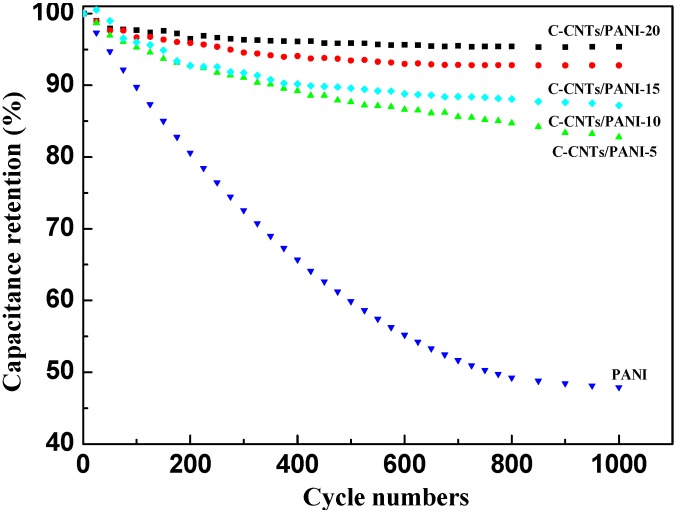
Cycling stability of the PANI and C-CNTs/PANI composite electrodes.

The cycling stability of the PANI and the C-CNTs/PANI composite electrodes were obtained after 1000 CV cycles at a scan rate of 0.1 V/s. It can be observed from Figure 9 that the composites exhibited significant higher specific cycling stability than the PANI; the C-CNTs/PANI-20 composite electrode retained 95.4% of its original capacitance after 1000 cycles, while the pure PANI retained only about 50%. Furthermore, the cycling stability of the C-CNTs/PANI composites were enhanced with the increased feeding ratio of C-CNTs. Their excellent cycling stability in comparison with the commonly reported CNTs/PANI composite electrodes is mainly due to the crosslinked carbon nanotubes network used for supporting PANI as conductive framework, as well as the covalent linkage between the C-CNTs and PANI [38]. Therefore, the C-CNTs/PANI composites showed great potential application in supercapacitors.

## 3. Experimental Section

### 3.1. Materials and Reagents

Multi-wall carbon nanotubes (L.MWCTs-60100, diameter: 60–100 nm, length: 5–15 μm, purity: ≥95%) were purchased from Shenzhen Nanotech Port Co. Ltd. (Shenzhen, China).

Aniline (Tianjin Fuchen Chemical Reagent Co., Tianjin, China) was distilled under reduced pressure before use. Ammonium peroxodisulfate (APS) (Tianjin Chemical Reagent Co., Tianjin, China), phenylenediamine (Tianjin Kaitong Chemical Reagent Co. Ltd., Tianjin, China), NaNO_2_ (Tianjin Chemical Reagent Sixth Factory, Tianjin, China), 1,4-benzoquinone (Sinopharm Chemical Reagent Co. Ltd., Shanghai, China), and other reagents were all analytical reagents and used without further purification. Deionized water was used throughout.

### 3.2. Synthesis of C-CNTs

The CNTs were firstly functionalized via a diazonium reaction [39]. Typically, 1.0 g phenylenediamine and 1.25 g NaNO_2_ were added into 10 mL *N*,*N*-dimethylfomamide (DMF). Ten milliliters of concentrated sulfuric acid were added into the mixture slowly in an ice water bath. After stirred at room temperature for 1 h, 0.3 g CNTs and 10 mL DMF were added, then the mixture was stirred and heated at 60 °C for 1 h. The product was centrifuged and washed thoroughly with DMF and ethanol.

Then the functionalized CNTs were crosslinked via coupling with 1,4-benzoquinone [22]. The functionalized CNTs were added into 60 mL ethanol solution which containing 15 mg 1,4-benzoquinone. The solution was ultrasonically dispersed for 30 min and then stirred at room temperature for 24 h. The C-CNTs were filtered and washed with ethanol and then vacuum dried at 40 °C for 12 h.

### 3.3. Synthesis of C-CNTs/PANI

The C-CNTs/PANI composites were prepared via the *in situ* chemical oxidative polymerization of aniline in the presence of the C-CNTs: 5.0 mL aniline and a certain amount of C-CNTs (feeding ratio to aniline of 0.05:1, 0.1:1, 0.15:1, or 0.2:1) were added into 26.3 mL 1.0 moL/L hydrochloric acid, respectively. Then 5 mL ethanol was added and ultrasonicated for 30 min to disperse the C-CNTs. Five milliliters of aqueous solution containing APS (molar ratio to aniline of 1:1) were added drop by drop to the above mixture over 20 min. The reaction vessel was maintained at room temperature for 6 h. The products were filtered and washed with a large amount of distilled water and then ethanol, followed by vacuum drying at 45 °C overnight. The products were denoted as C-CNTs/PANI-5, C-CNTs/PANI-10, C-CNTs/PANI-15, or C-CNTs/PANI-20 with the C-CNTs feeding ratio to aniline of 0.05:1, 0.1:1, 0.15:1, or 0.2:1, respectively.

For comparison, the pure PANI was synthesized with the method above without C-CNTs.

### 3.4. Characterizations and Testing

Transmission electron microscopy (TEM) measurements were carried out on a JEM-1230 transmission electron microscope (JEOL, Tokyo, Japan) operated at an accelerating voltage of 100 kV. The samples were dispersed in water and dropped onto the Cu grids covered with a perforated carbon film, followed by solvent evaporation in air at room temperature.

Fourier transform infrared (FT-IR) measurements (Impact 400, Nicolet, Waltham, MA, USA) were carried out in the range of 400−4000 cm^−1^ with a resolution of 4 cm^−1^ with the KBr pellet method.

Thermogravimetric analysis of the samples were characterized by a Diamond TG thermal analyzer from 30 to 800 °C at a heating rate of 10 °C/min in nitrogen atmosphere.

The electrical conductivities of the copolymer powders were measured using a RTS-2 four-point probe conductivity tester (Guangzhou four-point probe Technology Co., Ltd, Guangdong, China) at ambient temperature. The pellet was obtained by subjecting the powder to a pressure of about 20 MPa. The value is an average of at least three measurements.

The electrochemical properties were performed including galvanostatic charge/discharge (GCD), cyclic voltammetry (CV), electrochemical impedance spectrum (EIS) and cycling stability by a CHI660E electrochemical workstation in 1.0 moL/L H_2_SO_4_ aqueous electrolyte using a three-electrode setup. The C-CNTs/PANI composites served as the working electrode; the saturated calomel electrode (SCE) and platinum foil electrodes were used as the reference and counter electrodes, respectively. All tests (except for EIS) were carried out from −0.2–0.8 V (*vs.* SCE). The EIS of different electrodes were carried out at an open circuit potential of 0.4 V over a frequency range of 10^−2^−10^5^ Hz. The specific capacitance of the nanocomposites can be calculated from the CV curves according to the following equation:

*C_m_ = ∫IdV/(v△mV)*

where *I* means the response current density (A/g) in CV test, *V* is the potential (V), *v* is the scan rate (mV/s), and *m* is the total mass of the nanocomposites (g).

## 4. Conclusions

In summary, pristine commercial multi-walled carbon nanotubes were covalently crosslinked as the 3-D framework for polyaniline, which was grafted and coated via facile *in* *situ* chemical oxidative polymerization. Due to the highly conductive 3-D network of the crosslinked carbon nanotubes (C-CNTs) and the covalent linkage between the C-CNTs and PANI, the C-CNTs/PANI composites possessed higher electrical conductivity and specific capacitance than the pure PANI, with the maximum values of 12.12 S/cm and 298.4 F/g at a scan rate of 10 mV/s in 1.0 moL/L H_2_SO_4_ solution. Furthermore, the electrochemical cycling stability of the pseudocapacitive material has been significantly enhanced. After 1000 cycles at a scan rate of 0.1 V/s, the C-CNTs/PANI-20 composite electrode, prepared with the feeding ratio of the C-CNTs to aniline of 20%, retained more than 95% of its original capacitance. Therefore the C-CNTs/PANI composites based on the highly conductive 3-D framework showed exciting potential as high performance supercapacitors.
